# Ultrasound texture analysis: Association with lymph node metastasis of papillary thyroid microcarcinoma

**DOI:** 10.1371/journal.pone.0176103

**Published:** 2017-04-18

**Authors:** Soo-Yeon Kim, Eunjung Lee, Se Jin Nam, Eun-Kyung Kim, Hee Jung Moon, Jung Hyun Yoon, Kyung Hwa Han, Jin Young Kwak

**Affiliations:** 1 Department of Radiology, Severance Hospital, Research Institute of Radiological Science, Yonsei University College of Medicine, Seoul, Korea; 2 Department of Computational Science and Engineering, Yonsei University, Seoul, Korea; Universidade do Porto Faculdade de Medicina, PORTUGAL

## Abstract

This retrospective study aimed to evaluate whether ultrasound texture analysis is useful to predict lymph node metastasis in patients with papillary thyroid microcarcinoma (PTMC).

This study was approved by the Institutional Review Board, and the need to obtain informed consent was waived. Between May and July 2013, 361 patients (mean age, 43.8 ± 11.3 years; range, 16–72 years) who underwent staging ultrasound (US) and subsequent thyroidectomy for conventional PTMC ≤ 10 mm between May and July 2013 were included. Each PTMC was manually segmented and its histogram parameters (Mean, Standard deviation, Skewness, Kurtosis, and Entropy) were extracted with Matlab software. The mean values of histogram parameters and clinical and US features were compared according to lymph node metastasis using the independent t-test and Chi-square test. Multivariate logistic regression analysis was performed to identify the independent factors associated with lymph node metastasis. Tumors with lymph node metastasis (*n* = 117) had significantly higher entropy compared to those without lymph node metastasis (*n* = 244) (mean±standard deviation, 6.268±0.407 vs. 6.171±.0.405; *P* = .035). No additional histogram parameters showed differences in mean values according to lymph node metastasis. Entropy was not independently associated with lymph node metastasis on multivariate logistic regression analysis (Odds ratio, 0.977 [95% confidence interval (CI), 0.482–1.980]; *P* = .949). Younger age (Odds ratio, 0.962 [95% CI, 0.940–0.984]; *P* = .001) and lymph node metastasis on US (Odds ratio, 7.325 [95% CI, 3.573–15.020]; *P* < .001) were independently associated with lymph node metastasis. Texture analysis was not useful in predicting lymph node metastasis in patients with PTMC.

## Introduction

Papillary thyroid microcarcinoma (PTMC) is defined as thyroid cancer of size 1cm or smaller [1]. With the widespread use of ultrasound (US), PTMC has been detected more frequently worldwide [2]. Most PTMCs show an indolent nature and excellent outcomes following surgery [1,3–8]. Furthermore, a series of observational trials was able to reveal that most PTMCs do not progress to clinical disease on follow-up, thus concluding that PTMCs can be observed without immediate surgery [3,9,10]. However, a small number of PTMCs can present unfavorable features such as cervical lymph node or distant metastasis at diagnosis, and a few of PTMCs also have been reported to have aggressive outcomes such as loco-regional or distant recurrence following surgery [1,7,8]. The incidence of central lymph node metastasis was reported from 30% to 65% [11–14], and the incidence of lateral lymph node metastasis was reported from 3% to 44.5% in patients with PTMC [11,15]. The presence of lymph node metastasis has been reported to be the most powerful predictor of recurrence [16].

High-resolution ultrasound (US) is widely used in the preoperative evaluation of differentiated thyroid cancer [1]. Previous studies reported that several US features such as upper pole location, >25% contact with the adjacent thyroid capsule (e.i. suspicion of extrathyroidal extension on US), presence of calcifications, and ill-defined margin were associated with lateral lymph node metastasis in patients with PTMC [17–19]. However, evaluation with US is inherently limited as it is a subjective and operator-dependent diagnostic technique [20,21].

Texture analysis quantitatively evaluates spatial variation and distribution of gray levels within a targeted region of interest (ROI), which may enable a more objective and detailed assessment of lesion characteristics than visual analysis by human observers [22–26]. In recent studies [23,27], texture analysis did not improve diagnostic performance to differentiate benign and malignant thyroid nodules, while subjective analysis performed by radiologists using gray-scale US images had the best diagnostic performance. To our knowledge, there has been no study investigating whether texture analysis is useful in predicting lymph node metastasis in patients with PTMC.

If a texture parameter derived from preoperative staging US is associated with lymph node metastasis, it may be used to determine the extent of surgery and to predict prognostic outcomes. Accordingly, the purpose of this study was to investigate whether texture analysis is useful to predict lymph node metastasis in patients with PTMC.

## Materials and methods

The Institutional Review Board of Yonsei University College of Medicine, Severance Hospital, Seoul, Korea approved this retrospective study, and the requirement for informed consent was waived. Written informed consent for US-guided fine needle aspiration (US-FNA) and surgery was obtained from all patients prior to procedures.

### Patients

Between May and July 2013, 552 consecutive patients with US-FNA or core needle biopsy-proven PTC underwent preoperative staging US and subsequent thyroidectomy in our institution. Of these patients, 173 patients with PTC >10mm, 17 patients with follicular variant PTMC and one patient with oncocytic variant PTMC were excluded. Finally, a total of 361 patients with conventional PTMC confirmed by surgery were included. Among them, 85.6% (309 of 361) underwent FNA or core-needle biopsy at outside hospitals and 14.4% (52 of 361) underwent FNA at our institution. In our institution, during the study period, US-FNA was performed for thyroid nodules ≥ 5 mm with at least one suspicious US feature (marked hypoechogenicity, irregular or microlobulated margin, microcalcification, and taller-than-wide shape) according to previously published criteria [28]. Of 361 patients, 192 underwent total or near-total thyroidectomy, and 169 underwent lobectomy. The mean age of the 361 patients was 43.8±11.3 years (range, 16–73 years; 279 women [mean age, 43.9±11.5 years; range, 16–73 years] and 82 men [mean age, 43.6±10.3 years; range, 28–71 years]). One patient had gross extrathyroidal extension invading the strap muscle (Stage T3b according to the 8^th^ edition of the AJCC) [29]. No patient was found with gross extrathyroidal extension beyond the thyroid capsule invading the subcutaneous soft tissue, larynx, trachea, esophagus, or recurrent laryngeal nerves (Stage T4a), prevertebral fascia or encasing the carotid artery or mediastinal vessels (Stage T4b). Ninety eight patients had central lymph node metastasis only, 3 patients had lateral lymph node metastasis only, and 16 patients had both central and lateral lymph node metastasis. Overall, 117 (32.4%) patients had central and/or lateral lymph node metastasis. All patients were assessed with a low TNM stage (I-II) according to 8^th^ edition of the AJCC [29].

### Preoperative staging US

Preoperative staging US was performed prospectively by one of eight radiologists dedicated to thyroid imaging (four fellows with 1 or 2 years of experiences and five faculties with 6 to 15 years of experiences). We used one US scanner (iU22, Philips Medical Systems, Bothell, WA) with a 5-to 12-MHz linear transducer, and the presets of the scanner including gain and time-gain compensation were uniformed and these settings were not altered during the study period. All index tumors were routinely evaluated and captured in both transverse and longitudinal planes. The greatest dimension of the index tumor on US was measured for T staging. When the index tumor was limited within the thyroid, tumors ≤1cm were classified as T1a, those >1cm but ≤2cm as T1b, those >2cm but ≤4cm as T2, and those >4cm as T3 [29]. If the tumor of any size had gross extrathyroidal extention invading only strap muscles (sternohyoid, sternothyroid, or omohyoid muscles), the tumor was considered as T3b [29]. If the tumor of any size had gross extrathyroidal extention invading subcutaneous soft tissue, larynx, trachea, esophagus, or recurrent laryngeal nerve, the tumor was considered as T4a [29]. If the tumor of any size had gross extrathyroidal extension invading prevertebral fascia or encasing the carotid artery or mediastinal vessels, the tumor was considered as T4b [29]. The location of the index tumor was divided into three regions by height (upper, middle, or lower). The presence and type of calcifications within the index tumor were evaluated (no calcifications, macrocalcifications, or microcalcifications [includes nodules with both micro- and macrocalcifications]). Lymph nodes with at least one suspicious US feature (focal or diffuse hyperechogenicity, presence of internal calcification, cystic change, round shape, and chaotic or peripheral vascularity on Doppler US) were regarded as pathologic lymph nodes, and underwent US-FNA [30]. US-FNA was performed using a 23-gauge needle attached to a 2ml disposable plastic syringe. A small portion of the aspirated materials was rinsed with saline, and the washout was submitted for thyroglobulin measurement. N staging was as follows: N0, no evidence of lymph node metastasis, N1a, suspicious lymph nodes for metastasis in the ipsilateral or contralateral central compartment or both; N1b, suspicious lymph nodes for metastasis in the ipsilateral or contralateral lateral compartment or both. The staging US findings were prospectively recorded in the radiologic reports.

### Thyroid surgery and pathologic diagnosis

In our institution, total thyroidectomy is performed in cases with extrathyroidal extension, bilaterality, or lymph node metastasis found on preoperative or intraoperative findings. Prophylactic bilateral central compartment lymph node dissection is routinely performed in patients with total thyroidectomy, and prophylactic unilateral central compartment dissection is routinely performed in patients with hemithyroidectomy. Lateral compartment dissection is performed only when lateral lymph node metastasis is diagnosed on preoperative US-FNA or on an intraoperative frozen section. Central compartment dissection includes paratracheal, pretracheal, and prelaryngeal lymph nodes, and lateral compartment dissection includes lymph nodes at level 2, 3, 4, and anterior 5.

Extrathyroidal extension, central and/or lateral lymph node metastasis status, and TNM stages according to the 8^th^ edition of the AJCC [29] were reviewed based on the original pathological reports without the authors having knowledge of US features.

### Texture analysis

For texture analysis, first, a representative transverse or longitudinal US image was selected for each tumor. The representative US images that were previously captured by the radiologist who prospectively performed the US, were used. In our practice, the representative transverse image was captured when the tumor had the largest diameter, and the representative longitudinal image was captured when the tumor was at a right vertical angle to the representative transverse image. A manual segmentation for all cases was retrospectively performed by a dedicated thyroid radiologist (S.J.N. with 2 years of experience in thyroid US imaging). A region of interest was delineated around the boundary of the index tumor. To evaluate the interobserver variability for the histogram parameters, another thyroid radiologist (S.Y.K. with 2 years of experience in thyroid US imaging) independently performed the manual segmentation of the seventy tumors using the same US images.

**Fig 1:**
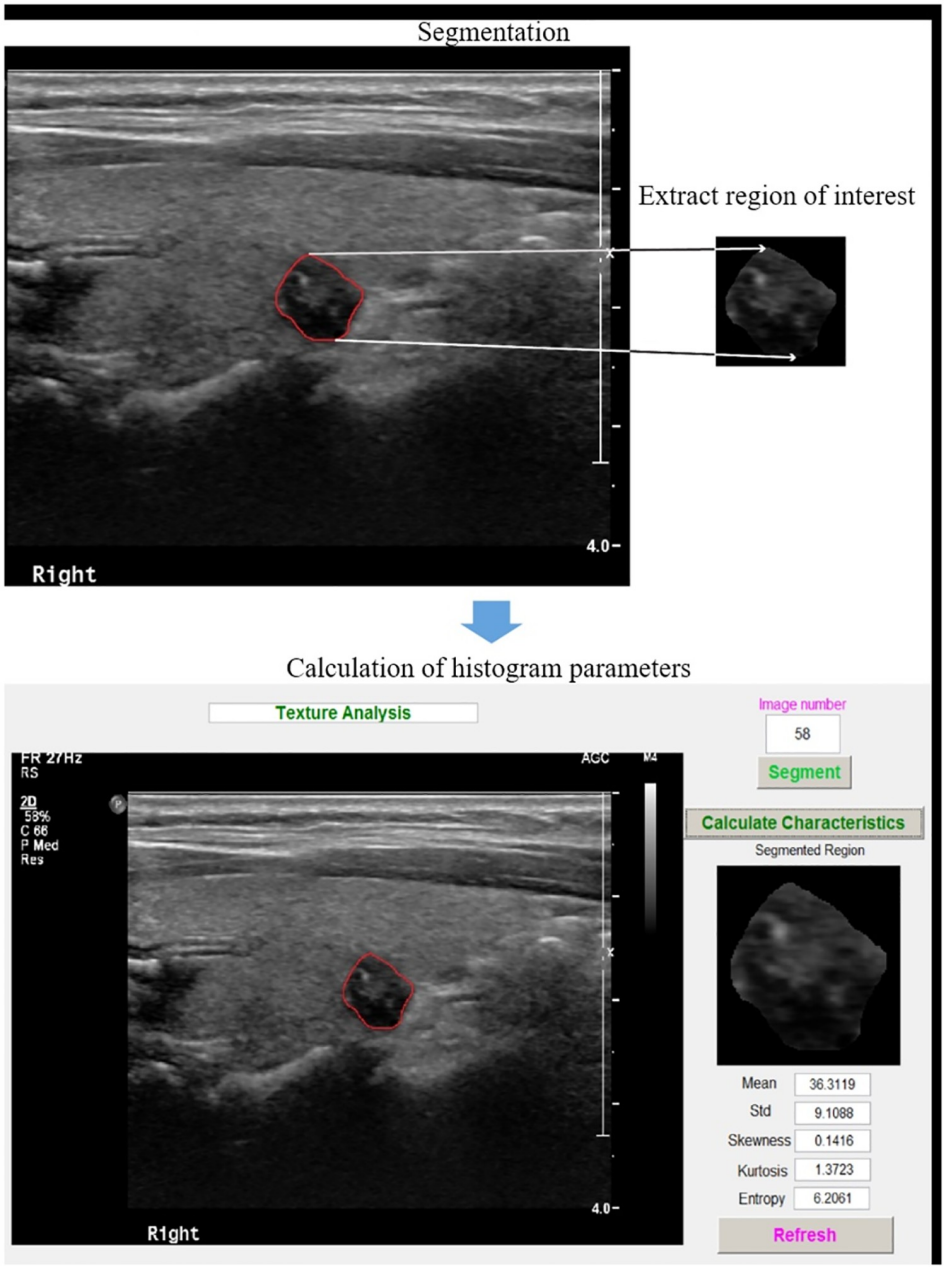
The process of the texture analysis. The segmentation of thyroid cancers was manually conducted and then histogram parameters were automatically calculated.

After the tumor was segmented, histogram parameters were calculated and extracted automatically (Fig 1). Histogram parameters included Mean, Standard deviation, Skewness, Kurtosis, and Entropy [23]. Mean was defined as the average value of pixel intensity (i.e. echogenicity of US) ranging from 0 (black on US) to 255 (white on US); Standard deviation as the standard deviation of pixel intensity; Skewness as a measure of the distribution asymmetry about the mean; Kurtosis as a measure of the peakedness of the distribution. Higher values of kurtosis indicate a peaked distribution and lower values of kurtosis indicate a flat distribution); and Entropy as a measure of texture irregularity. Tumor segmentation and histogram analysis were performed using Matlab R2010a (MathWorks, Natick, Massachusetts).

### Statistical analysis

Differences in the mean values of histogram parameters according to the presence of lymph node metastasis (central and/or lateral)were compared using the independent t-test. Clinical and US features according to the presence of lymph node metastasis were compared using the Chi-square test. Multivariate logistic regression analysis were used to find clinical and US factors, and histogram parameters associated with lymph node metastasis. Variables with *P* values <0.05 indicating statistical significance on independent t-test and Chi-square test and age, sex, and nodule size on US were included in the multivariate logistic regression analysis. Interobserver variability for histogram parameters obtained from two sets of region of interest that were manually segmented by two radiologists were investigated using the intraclass correlation coefficient and categorized as follows: Intraclass correlation coefficients 0.0–0.2, slight agreement; 0.2–0.4, fair agreement; 0.4–0.6, moderate agreement; 0.6–0.8, good agreement; 0.8–1.0, excellent agreement [31]. All statistical analyses were performed with PASW Statistics software (version 20, IBM-SPSS). Values of *P* <0.05 were considered statistically significant.

## Results

Table 1 compared the mean values of the histogram parameters according to lymph node metastasis. Tumors with lymph node metastasis had significantly higher entropy compared to those without lymph node metastasis (mean±standard deviation [SD], 6.268±0.407 vs. 6.171±0.405; *P* = .035). No additional histogram parameters showed differences in mean values according to lymph node metastasis.

**Table 1:** Comparison of histogram parameters according to lymph node metastasis

Variables	Yes (n = 117)	No (n = 244)	*P* value
Mean	67.635±18.717	66.40±18.917	.560
Standard deviation	8.281±0.808	8.189±0.82	.313
Skewness	0.170±0.036	0.176±0.038	.200
Kurtosis	1.511±0.207	1.549±0.23	.134
Entropy	6.268±0.407	6.171±0.405	.035

Note.― Values given are mean±standard deviation.

Table 2 compared the clinical and US features according to lymph node metastasis. Pathologic lymph node metastasis was associated with younger age (*P* < .001), larger nodule size on US (*P* < .001), the presence of calcification on US (*P* = .046), and lymph node metastasis on US (*P* < .001).

**Table 2:** Comparison of clinical and ultrasonographic features according to lymph node metastasis.

Variables	Yes (n = 117)	No (n = 244)	*P* value
Age	40.5±10.4	45.5±11.4	< .001
Gender Men Women	29 (35.4) 88 (31.5)	53 (64.6) 191 (68.5)	.515
Size on US (mm)	8.4±3.3	7.0±3.0	< .001
Location on US Upper 1/3 Mid 1/3 Low 1/3	32 (35.2) 62 (35.0) 23 (24.7)	59 (64.8) 115 (65.0) 70 (75.3)	.185
Location on US Upper 1/3 Mid-low	32 (35.2) 85 (31.5)	59 (64.8) 185 (68.5)	.516
Calcification on US No Yes	57 (28.1) 60 (38.0)	146 (71.9) 98 (62.0)	.046
LNM on US No Yes	77 (25.0) 40 (75.5)	231(75.0) 13 (24.5)	< .001

Note.― Values are mean±standard deviation or numbers of patients (percentages). LNM = lymph node metastasis. US = ultrasound.

Table 3 demonstrated the multivariate logistic regression analysis results to identify factors associated with lymph node metastasis. Younger age (Odds ratio, 0.962 [95% confidence interval (CI), 0.940–0.984]; *P* = .001) and lymph node metastasis on US (Odds ratio, 7.325 [95% CI, 3.573–15.020]; *P* < .001) were independently associated with lymph node metastasis. Entropy was not associated with lymph node metastasis (Odds ratio, 0.977 [95% CI, 0.482–1.980]; *P* = 0.949).

**Table 3:** Multivariate logistic regression analysis for association between clinical and ultrasonographic features, histogram parameters and lymph node metastasis.

Variables	Odds ratio	95% CI	*P* value
Age	0.962	0.940–0.984	.001
Men	1.285	0.729–2.265	.385
Size on US	1.088	1.003–1.179	.042
Calcifications on US	1.213	0.692–2.128	.500
LNM on US	7.325	3.573–15.020	< .001
Entropy	0.977	0.482–1.980	.949

Note.― LNM = lymph node metastasis. US = ultrasound.

Table 4 demonstrated the results of interobserver variability for histogram parameters. All histogram parameters showed good or excellent agreement.

**Table 4:** Interobserver variability for histogram parameters.

Variables	Intraclass Correlation Coefficient	95% Confidence Interval	*P* value
Mean	0.949	0.920, 0.968	< .001
Standard deviation	0.770	0.654, 0.850	< .001
Skewness	0.806	0.705, 0.875	< .001
Kurtosis	0.851	0.770, 0.905	< .001
Entropy	0.815	0.529, 0.912	< .001

## Discussion

Our study found that texture analysis was not useful in predicting lymph node metastasis in patients with PTMC. None histogram parameters were independently associated with lymph node metastasis.

Several prior studies have reported that US features can be used to predict the prognosis of PTC [17–19,32–34]. PTC with suspicious US features more frequently had lymph node metastasis at diagnosis and recurrence than PTC without suspicious US features [32,34]. Moreover, higher number of suspicious US features were associated with lymph node metastasis in patients with PTC [33]. In a study by Kwak et al. [17], upper pole location, minimal extrathyroidal extension on US (> 25% contact with the adjacent capsule), and the presence of calcifications were the independent predictors of lateral lymph node metastasis in patients with PTMC. However, the limitation of US is well known that US is a subjective method and dependent on the experience of the performer [20,21]. Texture analysis has recently gained the interests in the radiologic field with the advantages of the objectiveness [22–27]. Accordingly, we examined whether texture analysis can predict the lymph node metastasis in patients with PTMC, but our results found that texture analysis cannot predict the lymph node metastasis.

In our study, the incidence of central and lateral lymph node metastasis was 31.6% (114 of 361) and 5.2% (19 of 361), respectively, which were within the reported ranges [11–15]. Cervical lymph node metastasis at diagnosis has been reported to be an independent predictive factor for recurrence in patients with PTMC [35]. In our study, younger age and lymph node metastasis on US were independently associated with lymph node metastasis. Young age <40 years old was independently associated with the novel appearance of lymph node metastasis during follow-up in an observational trial for PTMC [3]. In addition, young age <50 years old was independently associated with pathologic central lymph node metastasis in patients with PTMC who had no evidence of clinically central lymph node metastasis [36]. Our results also showed an independent association between younger age and lymph node metastasis, supporting these previous results [3,36]. By demonstrating an independent association between lymph node metastasis on US and lymph node metastasis, we confirmed that US can accurately predict lymph node metastasis [37,38]. Entropy is a measure of texture irregularity, and higher entropy represents increased heterogeneity within the region of interest [25,26]. Tumors with lymph node metastasis had higher entropy compared to those without lymph node metastasis, but entropy was not independently associated with the presence of lymph node metastasis.

There were several limitations to our study. First, this study was of retrospective design performed in a single institution, thus it may be subject to potential bias. Second, the lateral compartment was dissected only when lateral lymph node metastasis was diagnosed on preoperative US-FNA or on an intraoperative frozen section. Lymph nodes that were not dissected and did not show suspicious US features were presumed to be non-metastatic. Because follow-up was not performed in this study, metastatic lymph nodes may have been neglected. Third, inter-observer variability may exist among the eight radiologists who performed the staging US due to their different experience levels. Fourth, histogram parameters were obtained with manual segmentation by a radiologist; thus, the results can be subjectively influenced. Although manual segmentation showed good or excellent interobserver agreement, a reliable and robust automatic extraction technique should be developed to overcome this variability issue. Fifth, one representative transverse or longitudinal US image in which the tumor showed its largest diameter was analyzed for texture analysis. Using 3-dimentional volumetric US images which cover the whole tumor would be optimal for obtaining comprehensive information on the tumor.

In conclusion, texture analysis was not useful in predicting lymph node metastasis in patients with PTMC.
